# Safety Concerns or Adverse Effects as the Main Reason for Human Papillomavirus Vaccine Refusal: National Immunization Survey–Teen, 2008 to 2019

**DOI:** 10.1001/jamapediatrics.2021.1585

**Published:** 2021-06-28

**Authors:** Onyema Greg Chido-Amajuoyi, Rajesh Talluri, Sahil S. Shete, Sanjay Shete

**Affiliations:** 1 Department of Epidemiology, The University of Texas MD Anderson Cancer Center, Houston; 2 Department of Data Science, The University of Mississippi Medical Center, Jackson; 3 Department of Psychology and Counseling, The University of Texas at Tyler; 4 Division of Cancer Prevention and Population Sciences, The University of Texas MD Anderson Cancer Center, Houston; 5 Department of Biostatistics, The University of Texas MD Anderson Cancer Center, Houston

## Abstract

This secondary analysis assesses safety concerns and adverse effects as reported reasons for HPV vaccine refusal.

Vaccination against the human papillomavirus (HPV) is effective at preventing several squamous cell carcinomas, yet the population-level uptake of the vaccine remains low in the US. Several factors contribute to HPV vaccine hesitancy and refusal; of note, safety concerns rank consistently high as a reason for nonvaccination.^1,2^ The COVID-19 pandemic has brought to the forefront the fragility of public confidence in the safety of vaccines.^3^ Therefore, this study examines safety concerns or adverse effects of the HPV vaccine as the main reason for nonvaccination over an 11-year period.

## Methods

Data for this study were derived from the National Immunization Survey–Teen (NIS-Teen), spanning from 2008 to 2019. The NIS-Teen is a population-based survey of parents or guardians of adolescents aged 13 to 17 years in their household and of their primary care professionals. The methodology used for the NIS-Teen has been described previously.^4^ The NIS-Teen was approved by the National Center for Health Statistics research ethics review board. NIS-Teen data are deidentified and publicly available; therefore, the secondary data analysis conducted in this study was exempt from institutional review board approval and informed consent in accordance with the Common Rule and University of Texas MD Anderson Cancer Center policy.

The primary outcome was reporting of safety concerns or adverse effects as the main reason for intention to refuse HPV vaccination for adolescents by their parent or guardian. This outcome was derived as a response to the survey question, “What is the MAIN reason teen will not receive HPV shots in the next 12 months?” among those who had not received any HPV vaccine and had no clear intention of receiving the vaccine. Joinpoint software version 4.8.0.1 (National Cancer Institute) was used to evaluate safety concerns and adverse effects over the study period. Weighted prevalence of safety concerns or adverse effects as the main reason for HPV nonvaccination were estimated for the overall population and by sociodemographic characteristics using R version 4.0.3 (The R Foundation). Tests were 2-tailed and significance was set at *P* < .05.

## Results

Self-reports of safety concerns or adverse effects as the main reason for HPV vaccine refusal increased over the study period. The prevalence increased from 5.3% (95% CI, 4.4-6.5) in 2008 to 12.9% (95% CI, 12.0-13.9) in 2015 with a slope of 0.9% increase per year. However, the prevalence substantially increased from 12.9% (95% CI, 12.0-13.9) in 2015 to 26.2% (95% CI, 24.3-28.2) in 2019 with a slope of 3.5% increase per year. The change in slope before and after 2015 was statistically significant (0.9% vs 3.5%; difference, 2.6%; 95% CI, 0.7-4.6; *P* = .03) (Figure). Throughout the study period, higher rates of safety concerns or adverse effects as the main reason for nonvaccination were reported by non-Hispanic White parents or guardians and by parents or guardians of teenaged girls (Table). From 2008 to 2013, mothers who were college graduates had rates of reporting safety concerns or adverse effects comparable with those among mothers with less than 12 years of education. However, from 2014 to 2019, there was a statistically significant increase in the reporting of safety concerns or adverse effects as the main reason for HPV nonvaccination by mothers with college degrees compared with those with less than 12 years of education (eg, 28.3% [95% CI, 25.5-31.2] among mothers with college degrees vs 13.7% [95% CI, 9.5-19.4] among mothers with less than 12 years of education in 2019) (Table).

**Figure.**
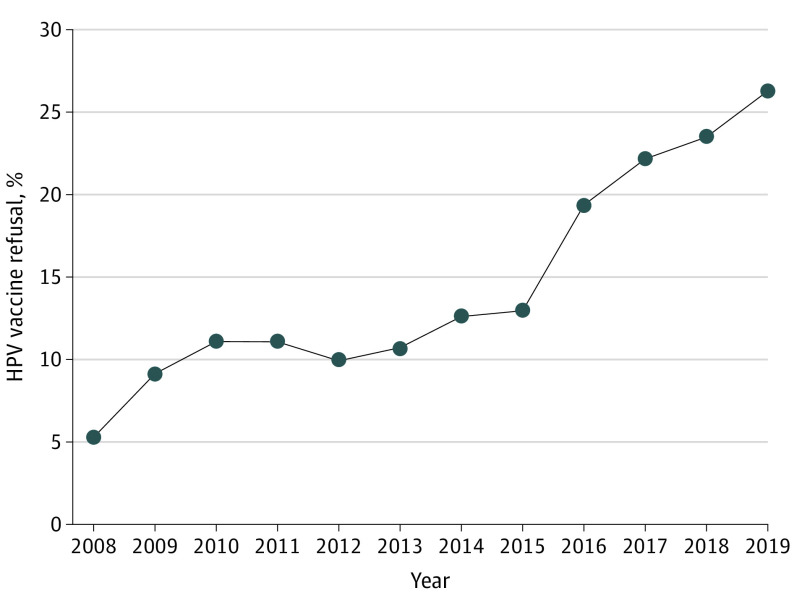
Prevalence of Safety Concerns or Adverse Effects as the Main Reason for Human Papillomavirus (HPV) Vaccine Refusal: National Immunization Survey–Teen, 2008 to 2019

**Table.** Prevalence of Safety Concerns or Adverse Effects as the Main Reason for Human Papillomavirus (HPV) Vaccine Refusal by Sociodemographic Characteristics: National Immunization Survey–Teen, 2008 to 2019

Characteristic	% (95% CI)											
	2019	2018	2017	2016	2015	2014	2013	2012	2011	2010	2009	2008
Total, No. (%; 95% CI)	1973 (26.2; 24.3-28.2)	2013 (23.4; 21.9-25.1)	2169 (22.1; 20.8-23.5)	2065 (19.3; 18.1-20.6)	1542 (12.9; 12.0-13.9)	1387 (12.6; 11.5-13.8)	1085 (10.7; 9.7-11.8)	1215 (9.9; 9.0-10.9)	1939 (11.1; 10.3-11.8)	1881 (11.1; 10.3-12.0)	519 (9.1; 8.1-10.3)	276 (5.3; 4.4-6.5)
Sex												
Male^a^	23.2 (20.9-25.8)	19.9 (18.0-21.9)	19.3 (17.6-21.1)	15.5 (14.1-16.9)	10.5 (9.4-11.7)	8.4 (7.2-9.8)	7.5 (6.4-8.7)	5.9 (5.1-6.8)	7.1 (6.4-7.9)	5.0 (4.4-5.6)	NA	NA
Female	30.0 (27.1-33.2)	27.4 (24.9-30.1)	25.7 (23.7-27.8)	24.3 (22.3-26.5)	16.4 (14.9-18.1)	18.7 (16.7-20.9)	15.6 (13.7-17.7)	16.8 (14.8-18.9)	18.6 (17.1-20.2)	22.7 (20.7-24.9)	9.1 (8.1-10.3)	5.3 (4.4-6.5)
Race/ethnicity												
Non-Hispanic White	29.7 (27.4-32.2)	26.7 (24.8-28.8)	24.4 (22.9-25.9)	21.4 (20.0-22.9)	15.0 (13.8-16.2)	13.1 (11.9-14.5)	11.7 (10.5-13.1)	10.1 (9.2-11.1)	11.4 (10.6-12.3)	11.0 (10.2-12.0)	10.3 (8.9-11.8)	5.7 (4.5-7.2)
Non-Hispanic Black	19.6 (14.7-25.7)	14.0 (10.6-18.1)	15.6 (11.9-20.3)	17.3 (13.2-22.3)	8.4 (6.2-11.3)	10.6 (7.7-14.5)	8.8 (6.4-11.8)	10.2 (7.5-13.7)	10.8 (8.7-13.4)	10.6 (8.7-12.8)	7.1 (5.0-10.2)	6.8 (4.1-11.0)
Hispanic	20.9 (16.5-26.3)	17.4 (13.9-21.5)	16.9 (13.3-21.3)	13.7 (10.9-17.1)	9.9 (7.9-12.4)	11.9 (8.6-16.2)	8.3 (6.0-11.5)	10.4 (7.7-13.9)	11.5 (9.3-14.2)	11.8 (8.8-15.6)	7.2 (4.7-10.9)	3.0 (1.6-5.5)
Multiple/other^b^	21.4 (17.1-26.6)	25.8 (20.2-32.4)	23.6 (19.4-28.4)	17.2 (13.8-21.3)	9.6 (7.3-12.6)	12.6 (8.6-18.1)	10.1 (7.2-14.1)	7.4 (4.3-12.3)	7.7 (5.9-10.0)	10.9 (8.6-13.8)	7.2 (4.3-11.8)	3.7 (2.1-6.6)
Maternal education, y												
<12	13.7 (9.5-19.4)	11.8 (8.6-15.9)	8.3 (6.2-11.1)	7.7 (5.6-10.6)	6.7 (4.8-9.4)	5.8 (3.7-9.0)	9.6 (6.2-14.5)	4.4 (2.9-6.7)	10.9 (8.1-14.6)	8.3 (5.8-11.6)	9.8 (6.1-15.3)	3.7 (2.1-6.4)
12	21.4 (17.6-25.8)	21.2 (17.9-25.0)	18.7 (15.6-22.3)	16.5 (14.0-19.3)	9.2 (7.6-11.2)	11.4 (9.1-14.2)	10.4 (8.4-12.9)	9.7 (7.6-12.2)	9.4 (8.1-11.0)	10.7 (9.3-12.4)	8.3 (6.3-10.8)	5.5 (3.5-8.3)
>12, Non–college graduate	30.4 (26.8-34.3)	25.3 (22.5-28.3)	23.7 (21.4-26.2)	20.9 (18.8-23.2)	14.9 (13.1-16.8)	14.8 (12.6-17.3)	11.3 (9.7-13.2)	11.5 (9.9-13.4)	11.9 (10.5-13.4)	12.2 (10.4-14.2)	9.6 (7.8-11.7)	5.4 (3.9-7.4)
College graduate	28.3 (25.5-31.2)	25.5 (23.0-28.1)	25.5 (23.5-27.6)	22.1 (20.2-24.2)	14.7 (13.2-16.3)	12.9 (11.3-14.8)	10.7 (9.1-12.5)	10.0 (8.7-11.5)	11.5 (10.4-12.7)	11.3 (10.1-12.6)	9.3 (7.7-11.1)	5.7 (4.1-7.9)
Poverty status (family income)^c^												
Below poverty level	18.9 (13.0-26.6)	21.9 (17.0-27.9)	16.1 (12.2-20.9)	12.7 (9.9-16.0)	11.4 (9.1-14.2)	10.4 (7.9-13.7)	11.0 (8.2-14.7)	9.3 (6.6-13.1)	10.1 (8.1-12.6)	9.2 (7.0-12.0)	8.9 (5.8-13.3)	1.9 (1.0-3.7)
Above poverty level (<$75 000)	27.2 (24.3-30.4)	24.0 (21.6-26.6)	22.0 (20.0-24.3)	20.2 (18.2-22.4)	11.8 (10.5-13.3)	12.7 (10.9-14.7)	10.9 (9.3-12.6)	9.1 (7.9-10.5)	11.2 (10.1-12.4)	11.2 (10.1-12.4)	9.3 (7.7-11.1)	5.8 (4.4-7.5)
Above poverty level (>$75 000)	28.2 (25.6-30.9)	23.7 (21.5-26.0)	24.3 (22.4-26.4)	20.4 (18.8-22.2)	14.3 (12.9-15.9)	13.0 (11.4-14.8)	10.3 (8.8-11.9)	10.9 (9.6-12.3)	11.1 (10.1-12.3)	11.7 (10.4-13.1)	9.7 (8.0-11.6)	6.6 (4.7-9.2)

Abbreviation: NA, not applicable.

^a^ HPV vaccination data for male children were not obtainable in 2008 and 2009 because of the absence of sex-neutral vaccination at this time.

^b^ These categories were consolidated by the NIS Teen survey administrators after participants answered multiple open-ended questions regarding their race and ethnicity (https://www.cdc.gov/vaccines/imz-managers/nis/downloads/NIS-Teen-Questionnaire-Q4-2019-508.pdf, 40-44). Race/ethnicity responses represented include American Indian, Alaska Native, Asian, Native Hawaiian, Pacific Islander, and those who selected more than one race.

^b^ Poverty level is defined according to the US Centers for Disease Control and Prevention National Immunization Survey–Teen based on family income and number of children (https://www.cdc.gov/vaccines/imz-managers/nis/downloads/NIS-TEEN-PUF19-CODEBOOK.pdf).

## Discussion

Overall, study findings suggest that safety concerns or adverse effects as the main reason for refusing HPV vaccination increased over the years. This finding has several important implications. First, given that concerns about vaccine safety are critical for vaccine confidence, rising safety concerns could negatively affect HPV vaccine uptake at the population level. Considering recent evidence of slowing routine HPV vaccination uptake,^5^ addressing safety concerns about vaccines should be of utmost public health importance.

Second, the findings of this study suggest that disinformation campaigns aimed at hampering vaccine trust are thriving. In the US, there has been a substantial rise of vaccine misinformation that has culminated in public mistrust in vaccines. The advent of social media and its exponential growth in popularity have served as a catalyst for spreading misinformation to a wider audience within the general public. In some instances, misinformation has also been supported by influential public and political figures. While our findings point to a need for widespread dissemination of educational programs within the general population,^6^ it is also crucial that public health agencies work with social media companies to develop campaigns to combat misinformation online. Lastly, physicians have a crucial frontline role to play in addressing vaccine hesitancy during parent-physician encounters.

Despite several strengths of our study, including using a rigorously designed nationally representative sample, our study is not without limitations, including low response rate and potential nonresponse bias. However, statistical adjustments, including standard weighting procedures, have been applied to account for such potential biases.

## References

[1] Hanson KE, Koch B, Bonner K, McRee AL, Basta NE. National trends in parental human papillomavirus vaccination intentions and reasons for hesitancy, 2010-2015. Clin Infect Dis. 2018;67(7):1018-1026. doi:10.1093/cid/ciy232 29596595 PMC6137113

[2] Thompson EL, Rosen BL, Vamos CA, Kadono M, Daley EM. Human papillomavirus vaccination: what are the reasons for nonvaccination among US adolescents? J Adolesc Health. 2017;61(3):288-293. doi:10.1016/j.jadohealth.2017.05.015 28842066

[3] Verger P, Dubé E. Restoring confidence in vaccines in the COVID-19 era. Expert Rev Vaccines. 2020;19(11):991-993. doi:10.1080/14760584.2020.1825945 32940574

[4] National Center for Immunization and Respiratory Diseases. National Immunization Survey–Teen: a user’s guide for the 2016 public-use data file. Accessed May 20, 2021. https://www.cdc.gov/vaccines/imz-managers/nis/downloads/NIS-TEEN-PUF16-DUG.pdf

[5] Chido-Amajuoyi OG, Talluri R, Wonodi C, Shete S. Trends in HPV vaccination initiation and completion within ages 9-12 years: 2008-2018. Pediatrics. Published online May 3, 2021. doi:10.1542/peds.2020-012765 33941585 PMC8785751

[6] Chido-Amajuoyi OG, Jackson I, Yu R, Shete S. Declining awareness of HPV and HPV vaccine within the general US population. Hum Vaccin Immunother. 2021;17(2):420-427. doi:10.1080/21645515.2020.1783952 32692632 PMC7899652

